# Role for a Lytic Polysaccharide Monooxygenase in Cell Wall Remodeling in Streptomyces coelicolor

**DOI:** 10.1128/mbio.00456-22

**Published:** 2022-03-31

**Authors:** Xiaobo Zhong, Le Zhang, Gilles P. van Wezel, Erik Vijgenboom, Dennis Claessen

**Affiliations:** a Molecular Biotechnology, Institute of Biology, Leiden University, Leiden, The Netherlands; Newcastle University; Harvard Medical School

**Keywords:** cell wall biosynthesis, LPMO, peptidoglycan, apical growth, morphology, glycan, cellulose, cell wall

## Abstract

Peptidoglycan is a major constituent of the bacterial cell wall and an important determinant for providing protection to cells. In addition to peptidoglycan, many bacteria synthesize other glycans that become part of the cell wall. Streptomycetes grow apically, where they synthesize a glycan that is exposed at the outer surface, but how it gets there is unknown. Here, we show that deposition of the apical glycan at the cell surface of Streptomyces coelicolor depends on two key enzymes, the glucanase CslZ and the lytic polysaccharide monooxygenase LpmP. Activity of these enzymes allows localized remodeling and degradation of the peptidoglycan, and we propose that this facilitates passage of the glycan. The absence of both enzymes not only prevents morphological development but also sensitizes strains to lysozyme. Given that lytic polysaccharide monooxygenases are commonly found in microbes, this newly identified biological role in cell wall remodeling may be widespread.

## INTRODUCTION

Bacteria can thrive successfully in almost all environments. Part of their success is attributed to the presence of a cell wall that provides protection against environmental insults. A major component of the bacterial cell wall is peptidoglycan (PG), which is a layered mesh of glycan strands composed of alternating *N*-acetylglucosamine (GlcNAc) and *N*-acetylmuramic acid (MurNAc) moieties (1). These glycan strands are cross-linked via short peptide bridges, thereby creating a robust structure. In addition to PG, the cell wall often comprises other macromolecules, including teichoic acids and capsular polysaccharides (CPs) (2, 3). Synthesis and assembly of all these components must be tightly regulated in space and time to ensure that the cell’s integrity is not compromised.

Streptomycetes are Gram-positive bacteria with a multicellular lifestyle (4). They are producers of a wide variety of bioactive natural products, including over half of all clinical antibiotics (5). Unlike unicellular bacteria, streptomycetes grow as long, branching filaments (called hyphae) that collectively form a mycelial network. Interestingly, their cell wall architecture is complex and multilayered (6). New cell wall material is incorporated exclusively at the hyphal tips, via a process known as polar growth (7, 8). Such tips also produce glycans other than PG, which are positioned exterior of the PG layer (6). The two best-studied glycans are a *β*-(1-4)-glycan (also referred to as a cellulose-like glycan) and poly-*β*-(1-6)-*N*-acetylglucosamine (PNAG) (9, 10). These glycans play pivotal roles in morphological development. For instance, streptomycetes form reproductive aerial hyphae when nutrients become scarce, but this process is blocked when the cellulose-like glycan is absent (11, 12). Likewise, the absence of either PNAG or the cellulose-like glycan prevents the formation of auto-aggregated biofilm-like structures (called pellets) in liquid-grown environments (12). So far, little is known about how these glycans traverse the PG layer to become exposed at the cell surface.

**FIG 1:**
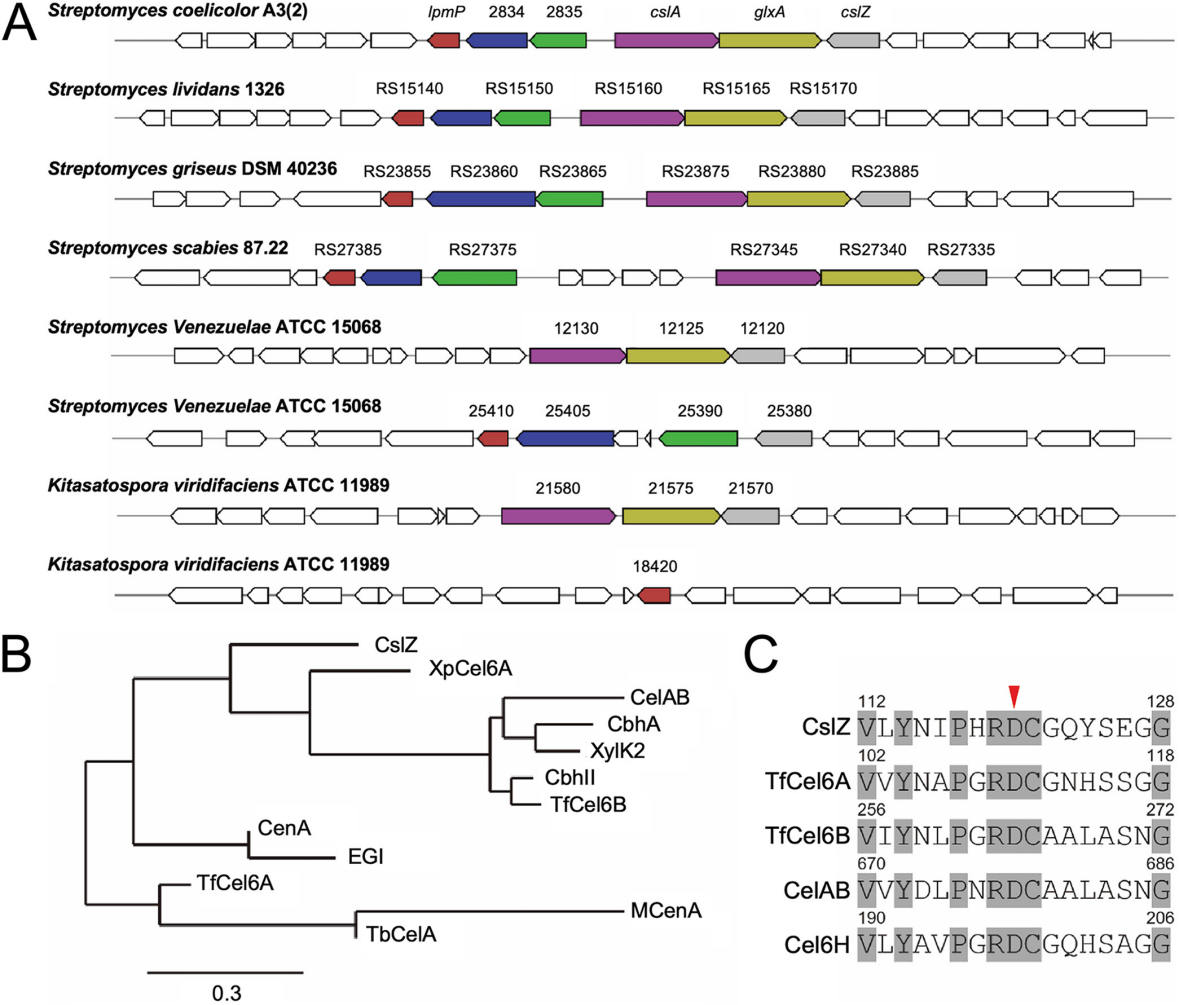
Comparative analysis of glycoside hydrolase family 6 proteins. (A) MultiGeneBlast output showing gene clusters of filamentous actinobacteria, which are homologous to the *cslA-glxA-cslZ* gene cluster of S. coelicolor involved in synthesis of a cellulose-like polymer. Clusters have a minimal identity of 30% and minimal sequence coverage of 25% to the S. coelicolor gene cluster. (B) Phylogenetic tree of members of the GH6 family, including CslZ (S. coelicolor), XpCel6A (Xylanimicrobium pachnodae), CelAB (Teredinibacter turnerae T7901), CbhA (Cellulomonas fimi ATCC 484), XylK2 (*Cellulosimicrobium* sp. HY-13), CbhII (*Streptomyces* sp. M23), TfCel6B (Thermobifida fusca YX), CenA (Mycobacterium tuberculosis H37Rv), EGI (Neisseria sicca SB), TfCel6A (Thermobifida fusca YX), McenA (Micromonospora cellulolyticum), and TbCelA (Thermobispora bispora), which were selected based on the availability of experimental data on their substrates. (C) Alignment of the catalytic centers of CslZ and other GH6s hydrolases, including TfCel6A, TfCel6B, CelAB, and Cel6H. The conserved residues in the catalytic centers are gray-colored and the key catalytic residue Asp is labeled with a red arrowhead. The full-length alignments of the GH6 domains are available in Fig. S2.

The cellulose-like polymer was identified over a decade ago and found to be produced at hyphal tips by the cooperative action of a cellulose synthase-like protein CslA and the copper radical oxidase GlxA (11–13). Transcription of *cslA* and that of *glxA* are coupled, and inactivation of either gene abolishes deposition of the cellulose-like glycan at hyphal tips (13). The *cslA-glxA* operon is followed by the divergently transcribed *cslZ*, which encodes a putative glucanase (see Fig. 1). This gene organization is conserved in most streptomycetes, suggesting that CslZ’s function perhaps relates to synthesis of the cellulose-like glycan (14). However, contrary to the absence of *cslA* or *glxA*, inactivation of *cslZ* in Streptomyces lividans has no clear effect on morphogenesis (12).

Upstream and in close proximity of *cslA-glxA-cslZ* lies a gene for a lytic polysaccharide monooxygenase (LPMO; SLI_3182/LPMO10E [15]), referred to here as *lpmP*. LPMOs are known to cleave polysaccharides through an oxidative mechanism and play a major role in carbon recycling in industry (16–18). Through random oxidation of polysaccharide substrates, LPMOs help to expose the well-organized microfibrils and increase their accessibility for other hydrolases. Consequently, these hydrolases can more efficiently degrade these polysaccharides (19–21). Notably, LPMO-encoding genes are ubiquitous in bacteria and fungi, although their biological roles have remained largely elusive. Only recently, LPMOs have been found to play roles in promoting Pseudomonas aeruginosa virulence (22), capturing copper in fungal meningitis (23), and degrading lignin (24, 25).

In this study, we demonstrate that the absence of both *lpmP* and *cslZ* prevents morphological development in Streptomyces coelicolor and makes the mycelium more sensitive to lysozyme. These phenotypes coincide with the inability of the double mutant to deposit the CslA-produced glycan at hyphal tips. Notably, this study shows that CslZ and LpmP can degrade PG. Taken together, these results show that LpmP and CslZ are crucial players involved in cell wall remodeling by facilitating localized PG degradation to enable deposition of a protective cellulose-like glycan on the cell surface. Given that LPMOs are ubiquitous in microbes, we anticipate that these enzymes more generally play important roles in cell wall remodeling.

## RESULTS

### Cooccurrence and clustering of genes involved in synthesis and degradation of glycans.

It was previously shown that *cslA* is required for synthesis of a cellulose-like glycan that is exposed at the cell surface of hyphal tips (11, 26). In most *Streptomyces* species, *cslA* is located in a conserved gene cluster, harboring *cslA*, *glxA*, and the divergently transcribed *cslZ*, with the latter encoding a putative glucanase (14) (Fig. 1A). CslZ is a putative lipoprotein (27), and BLAST analysis revealed that CslZ belongs to the glycoside hydrolase family 6 (GH6) proteins (accession number: WP_011028610.1). GH6 hydrolases cleave *β*-(1-4)-glycosidic bonds in polymers such as cellulose and also in other *β*-(1, 4)-glycans such as xylan or chitin (28, 29) (Fig. 1B, Table 1). CslZ lacks carbohydrate-binding modules (CBM) that some other members of the GH6 hydrolases possess (Fig. S1). Notably, the active site region of CslZ (residues 112 to 128) is strikingly similar to that of other GH6 family members and contains the catalytic residue Asp120, which is proposed as the key catalytic acid in the inverting catalytic mechanism (30, 31) (Fig. 1C, Fig. S2). These *in silico* analyses identify CslZ as a member of the GH6 family of hydrolases active on *β*-(1-4)-glycans.

**TABLE 1:** Hydrolases belonging to the GH6 family, including their substrates

Hydrolase	Organism	Substrate(s)	Reference
CslZ	Streptomyces coelicolor	Unknown	This study
XpCel6A	Xylanimicrobium pachnodae *DSM* 12657	Cellulose	52
CelAB	Teredinibacter turnerae	Cellulose, chitin	53
CbhA	Cellulomonas fimi *ATCC* 484	Cellulose	54
XylK2	*Cellulosimicrobium sp.* HY-13	Xylan	55
CbhII	*Streptomyces sp.* M23	Cellulose	56
TfCel6B	Thermobifida fusca	Cellulose	57
CenA	Cellulomonas fimi *ATCC* 484	Cellulose	58
EGI	Neisseria sicca *SB*	Cellulose acetate	59
TfCel6A	Thermobifida fusca	Cellulose	60
MCenA	Micromonospora cellulolyticum	Carboxymethyl cellulose	61
TbCelA	Thermobispora bispora	Cellobiose	62

10.1128/mbio.00456-22.1 FIG S1 Comparison of CslZ with other GH6 family members. Schematic overview of hydrolases belonging to the glycoside hydrolase family 6 (GH6). Shown are hydrolases from Streptomyces coelicolor (CslZ), Thermobifida fusca (TfCel6A, TfCel6B), Teredinibacter turnerae (CelAB), and an uncultured bacterium (Cel6H). The signal peptides (SP), GH6 domains, carbohydrate-binding modules (CBMs), cytosolic domain (CD), and transmembrane helices (TM) are indicated. Download FIG S1, TIF file, 0.2 MB. Copyright © 2022 Zhong et al. 2022 Zhong et al. https://creativecommons.org/licenses/by/4.0/ This content is distributed under the terms of the Creative Commons Attribution 4.0 International license.

10.1128/mbio.00456-22.2 FIG S2 Sequence alignment of GH6 domains. Sequence alignment of the GH6 domains of hydrolases from Streptomyces coelicolor (CslZ), Thermobifida fusca (TfCel6A, TfCel6B), Teredinibacter turnerae (CelAB), and an uncultured bacterium (Cel6H). Conserved residues are gray-colored, and the key catalytic residue Asp is labeled with a red arrowhead. Download FIG S2, TIF file, 1.3 MB. Copyright © 2022 Zhong et al. 2022 Zhong et al. https://creativecommons.org/licenses/by/4.0/ This content is distributed under the terms of the Creative Commons Attribution 4.0 International license.

Three genes, SCO2833 to 2835, are well conserved in streptomycetes and predominantly cluster with—and lie upstream of—*cslA-glxA-cslZ* (Fig. 1A). SCO2834 is a membrane protein that belongs to the so-called SPFH (stomatin, prohibitin, flotillin, and HflK/C) superfamily of proteins, which often associate with or form microdomains in membranes. SCO2835 is a putative membrane protein with a peptidoglycan-binding domain. LpmP (SCO2833) was shown to be a copper-dependent lytic polysaccharide monooxygenase (LPMO) active on chitin (15). Importantly, such LPMOs typically work in conjunction with hydrolytic enzymes to degrade recalcitrant polysaccharides (18, 32).

### CslZ and LpmP are required for morphological development in Streptomyces coelicolor.

**FIG 2:**
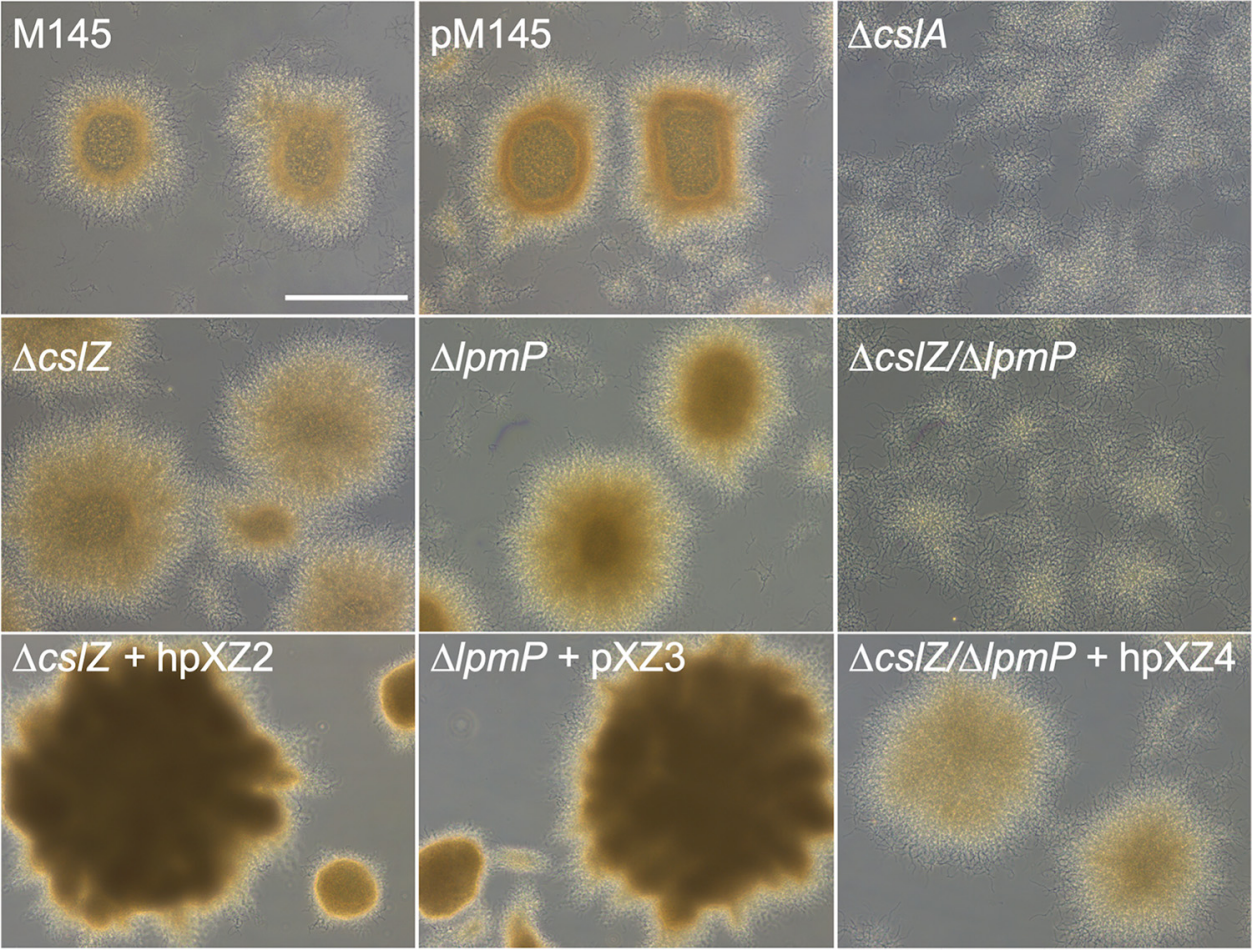
The absence of *lpmP* and *cslZ* affects mycelial morphology in S. coelicolor. Pellet morphology of strains lacking or overexpressing genes involved in glycan biosynthesis and degradation. Pellets were imaged after 48 h of growth in TSBS. The double mutant strain lacking *lpmP* and *cslZ* (*ΔcslZ/ΔlpmP*) is no longer able to form pellets and is phenotypically similar to the *cslA* mutant (*ΔcslA*). Reintroduction of both genes expressed from the constitutive *gapAp* promoter (plasmid hpXZ4) in the *ΔcslZ/ΔlpmP* double mutant restored wild-type pellet morphology. Pellets of the complemented single mutants expressing *cslZ* (plasmid hpXZ2) or *lpmP* (plasmid pXZ3) under the control of the constitutive *gapAp* promoter have a denser appearance compared to the wild-type strain. Pellets of the strain containing the empty pSET152 plasmid (pM145) were comparable to those of the wild type. Scale bar represents 100 μm.

To investigate the roles of CslZ and LpmP in morphogenesis, we first constructed a *cslZ* null mutant using plasmid pΔ*cslZ* (12). To do so, nucleotides +15 to +1011 relative to the translational start site of *cslZ* were replaced by an apramycin resistance marker. Furthermore, we inactivated *lpmP* using plasmid pXZ5 in the wild-type strain and in the Δ*cslZ* single mutant, yielding a markerless *lpmP* single mutant and an apramycin-resistant Δ*cslZ*/Δ*lpmP* double mutant (see Materials and Methods). Analysis of the Δ*cslZ* and Δ*lpmP* mutants in liquid media revealed that the morphology of the mycelial pellets was comparable to that of the mycelial pellets of the wild-type strain (Fig. 2). However, the constructed double mutant lacking *lpmP* and *cslZ* was no longer able to form pellets and was phenotypically similar to the Δ*cslA* mutant (Fig. 2). Reintroduction of both genes expressed from the constitutive *gapAp* promoter (33) in the Δ*cslZ*/Δ*lpmP* double mutant restored wild-type pellet morphology (Fig. 2). Furthermore, when *cslZ* (plasmid hpXZ2) or *lpmP* (plasmid pXZ3) was constitutively expressed in the respective single mutants (Fig. 2) or as an extra copy in a wild-type background (Fig. S3), denser pellets were obtained after 48 h. These results show that CslZ and LpmP together are required for pellet formation in *Streptomyces* and that in the absence of both proteins a synthetic phenotype becomes evident that is similar to the absence of CslA.

10.1128/mbio.00456-22.3 FIG S3 Constitutive expression of *lpmP* and *cslZ* affects pellet morphology and tip staining. (A) Constitutive expression of *cslZ* (plasmid pXZ2), *lpmP* (plasmid pXZ3), or both genes (plasmid pXZ4) in a wild-type background leads to pellets with a denser appearance after 48 h. (B) Tip staining of β-(1-4) glycans with calcofluor white is increased following expression of *cslZ* and *lpmP* from the constitutive *gapAp* promoter. Scale bars represent 200 μm (A), 100 μm (B), or 20 μm (insets in panel B). Download FIG S3, TIF file, 2.4 MB. Copyright © 2022 Zhong et al. 2022 Zhong et al. https://creativecommons.org/licenses/by/4.0/ This content is distributed under the terms of the Creative Commons Attribution 4.0 International license.

### Glycan deposition at hyphal tips is crucial for protection against lysozyme and depends on CslZ, LpmP, and CslA.

**FIG 3:**
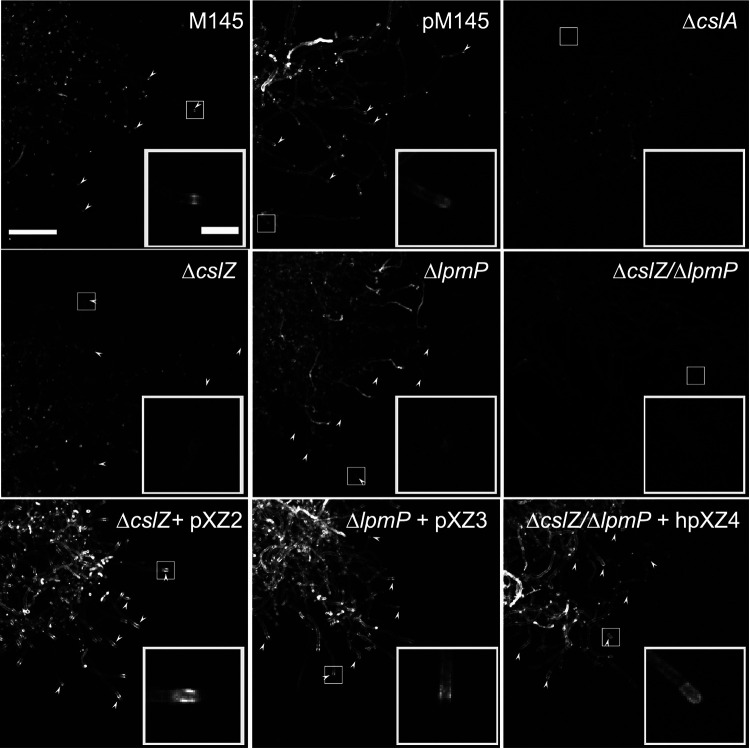
Deposition of the β-(1-4)-glycan at hyphal tips is abolished in the absence of LpmP and CslZ. Calcofluor white (CFW) staining was used to detect β-(1-4) glycans in S. coelicolor strains lacking genes involved in glycan biosynthesis and degradation. As expected, tip staining (arrowheads) is evident in the wild-type strain and control strain (*pM145*) and absent in the *ΔcslA* mutant (see insets). Tip staining is reduced in the *ΔcslZ* and the *ΔlpmP* single mutants but is absent in the *ΔlpmP/ΔcslZ* double mutant. Reintroduction of both genes expressed from the constitutive *gapAp* promoter (plasmid hpXZ4) in the *ΔcslZ/ΔlpmP* double mutant restored tip staining. The complemented single mutants expressing *cslZ* (plasmid hpXZ2) or *lpmP* (plasmid pXZ3) under the control of the constitutive *gapAp* show an increased staining compared to the wild-type (see also Fig. S4). Scale bars represent 100 μm (main images) and 20 μm (insets).

The nonpelleting phenotype of the Δ*cslZ/*Δ*lpmP* double mutant prompted us to investigate whether the glycan produced by CslA was still detectable at hyphal tips. To this end, we stained mycelium with calcofluor white, which binds to *β*-(1-4) glycans (11). Contrary to those of the wild-type strain, hyphal tips of the Δ*cslZ*/Δ*lpmP* double mutant no longer stained, a phenotype shared with the Δ*cslA* mutant. Reintroduction of both genes expressed from the constitutive *gapAp* promoter in the double mutant restored tip staining at hyphal tips (Fig. 3). Importantly, tip staining was also reduced in the single Δ*cslZ* or Δ*lpmP* mutants (Fig. 3, Fig. S4), indicating that CslZ and LpmP have direct roles in deposition of the glycan produced by CslA. Interestingly, when CslZ or LpmP were expressed from the constitutive *gapAp* promoter, tip staining was more pronounced (Fig. 3, Fig. S4). Altogether, these results show that CslZ and LpmP together are essential for glycan deposition at hyphal tips.

10.1128/mbio.00456-22.4 FIG S4 Quantitative analysis of the number of β-(1, 4) glycans present at hyphal tips of *Streptomyces* strains. Total fluorescence of calcofluor white-stained tips was determined in square regions of 15 μm by 15 μm. For each strain, 20 tips were measured. The total fluorescence in each strain was corrected for the fluorescence measured in the *cslA* mutant, which does not produce the β-(1, 4)-glycan. Fluorescence detected for the wild-type strain was set to 100%. Error bars represent the standard error of the mean. Download FIG S4, TIF file, 3.0 MB. Copyright © 2022 Zhong et al. 2022 Zhong et al. https://creativecommons.org/licenses/by/4.0/ This content is distributed under the terms of the Creative Commons Attribution 4.0 International license.

**FIG 4:**
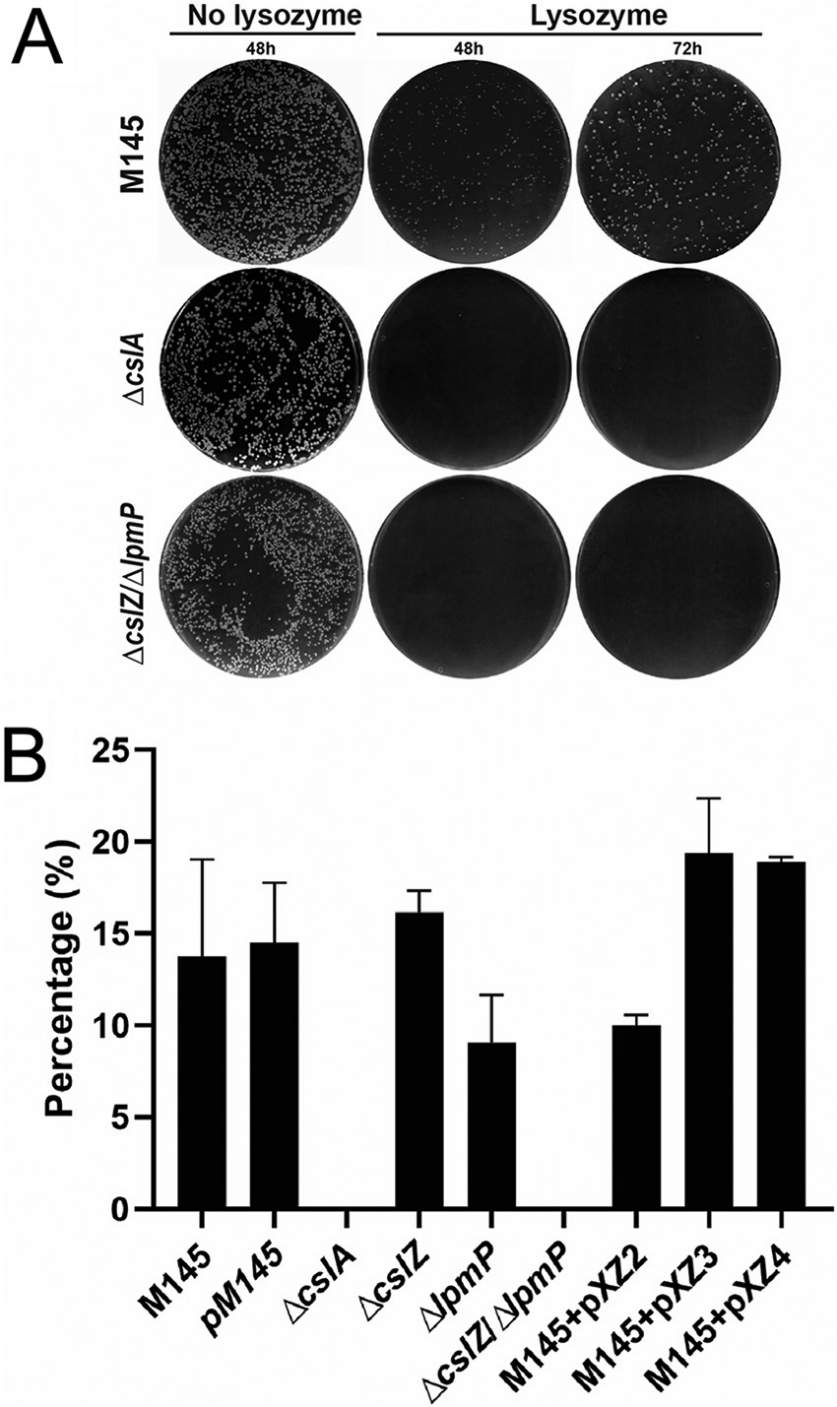
The absence of the CslA-produced polymer causes lysozyme sensitivity in S. coelicolor. (A) Growth of the wild-type strain, the *ΔcslA* mutant, and the *ΔlpmP/ΔcslZ* double mutant on plates with or without lysozyme (0.25 mg mL^−1^). No growth is observed for the *ΔcslA* mutant and the *ΔlpmP/ΔcslZ* double mutant on plates containing lysozyme. (B) Quantitative assessment of the relative number of CFU obtained following growth in the presence and absence of lysozyme. Percentages were determined by dividing the number of colonies on plates with 0.25 mg mL^−1^ lysozyme by the number of colonies on plates without lysozyme. The values represent the average of triplicate experiments. The error bars indicate the standard errors of the mean (*P* < 0.01).

Previous studies revealed that the CslA-produced glycan is located exterior to the PG layer, presumably providing protection during tip growth (6, 11, 34). To test this hypothesis, we exposed strains to a variety of cell wall-targeting agents. When the strains were grown in the presence of penicillin or ampicillin (acting on the synthesis of PG), no major differences in growth inhibition were observed between the wild-type strain and its mutants (Fig. S5). However, exposure to 0.25 mg mL^−1^ lysozyme (acting on intact PG) revealed a dramatically reduced viability of the Δ*cslA* strain and the Δ*cslZ*Δ*/lpmP* double mutant compared to that of the wild-type strain and the single mutants (Fig. 4). At lower lysozyme concentrations, it was evident that the Δ*cslZ*Δ*/lpmP* double mutant was more sensitive to lysozyme than the Δ*cslA* strain (Fig. S6). This suggests that the activities of CslZ and LpmP contribute to lysozyme protection not only via their role in glycan deposition. Altogether, these results show that presence of the cellulose-like glycan confers resistance to high levels of lysozyme and are consistent with the glycan being positioned exterior to the PG layer on the hyphal surface.

10.1128/mbio.00456-22.5 FIG S5 Antibiotic sensitivity of S. coelicolor strains lacking genes involved in the biosynthesis pathway of the CslA-produced polymer. Difco nutrient agar plates (25 mL) were overlaid with 2.5 mL of 0.5% nutrient agar containing 10^7^ spores of each strain. Whatman discs (6 mm, Sigma) were placed on top of the soft agar, after which 5 μL of ampicillin (left) or penicillin G (right) was applied to the discs. Bars indicate the inhibition zones (in mm) obtained after 48 h growth at 30°C. Inhibition zones were measured by ImageJ. Error bars indicate standard errors of the mean. Download FIG S5, TIF file, 2.9 MB. Copyright © 2022 Zhong et al. 2022 Zhong et al. https://creativecommons.org/licenses/by/4.0/ This content is distributed under the terms of the Creative Commons Attribution 4.0 International license.

10.1128/mbio.00456-22.6 FIG S6 Differences in lysozyme sensitivity between the Δ*cslA* and the Δ*cslZ*/Δ*lpmP* mutant strains. Quantitative assessment of the relative number of CFUs of *Streptomyces* mutant strains grown on plates with different concentrations of lysozyme. Percentages were determined by dividing the number of colonies on plates with lysozyme by the number of colonies on plates without lysozyme. No growth is observed for the *ΔcslA* mutant and the *ΔcslZ/ΔlpmP* double mutant on plates containing 250 μg mL^−1^ lysozyme. Unlike the double mutant, the *ΔcslA* mutant is able to grow on plates containing 10 μg mL^−1^ lysozyme. Reintroduction of the *cslZ* and *lpmP* genes constitutively expressed from the *gapAp* promoter appeared to restore lysozyme resistance at 2 μg mL^−1^ (*P = *0.085) but was unable to restore lysozyme resistance at higher lysozyme concentrations. The error bars indicate the standard errors of the mean. Download FIG S6, TIF file, 2.4 MB. Copyright © 2022 Zhong et al. 2022 Zhong et al. https://creativecommons.org/licenses/by/4.0/ This content is distributed under the terms of the Creative Commons Attribution 4.0 International license.

### CslZ is associated with the membrane and interacts with LpmP.

To localize CslZ, we produced a C-terminal FLAG-tagged version of CslZ in the wild-type strain. Western blotting revealed that the majority of CslZ was associated with the membrane, while a small fraction was detected in the cytoplasm (Fig. S7A). Elongation factor EF-Tu1, used as a control, was detected only in the cytoplasm (Fig. S7A). To investigate if CslZ and LpmP directly interact with each other, we performed a bacterial two-hybrid analysis (Fig. S7B). To this end, constructs were generated that produced C-terminal fusions of LpmP and CslZ (either with or without their signal sequences) to the T25 and T18 fragments of the adenylate cyclase, respectively. Cotransformation of these constructs revealed a robust interaction between CslZ and LpmP irrespective of the presence of the signal sequences (Fig. S7B). These data demonstrate that CslZ is a lipoprotein that strongly interacts with LpmP.

10.1128/mbio.00456-22.7 FIG S7 CslZ is predominantly localized at the membrane and interacts with LpmP. (A) Western blotting showing the detection of CslZ (FLAG-tagged) and EF-Tu1 in different fractions. T represents the total protein, whereas M and C represent the membrane and cytosolic fractions, respectively (see Materials and Methods). (B) Bacterial two-hybrid analysis demonstrating that CslZ and LpmP interact. CslZ and LpmP interact with each other either with (T25-LpmP_FL_/T18-CslZ_FL_) or without their signal sequences (T25-LpmP/T18-CslZ), as deduced from the blue-colored colonies. Cotransformants obtained with pKT25/pUT18c and pKT25-zip/pUT18C-zip were used as negative and positive controls, respectively. Download FIG S7, TIF file, 2.6 MB. Copyright © 2022 Zhong et al. 2022 Zhong et al. https://creativecommons.org/licenses/by/4.0/ This content is distributed under the terms of the Creative Commons Attribution 4.0 International license.

### LpmP binds to PG and facilitates PG hydrolysis.

**FIG 5:**
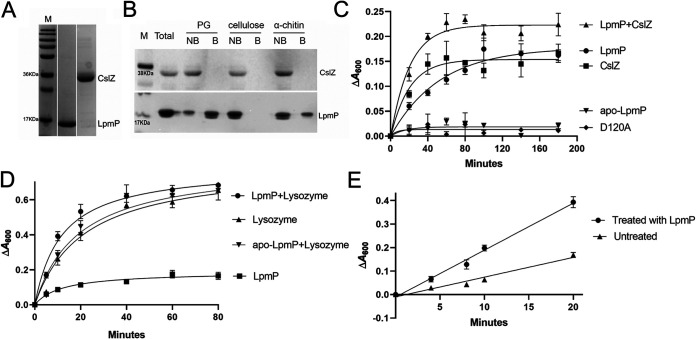
LpmP facilitates hydrolysis of peptidoglycan by lysozyme and CslZ. (A) SDS-PAGE gel showing purified LpmP (18.4 kDa) and CslZ (31.9 kDa) heterologously produced in E. coli. (B) *In vitro* binding assays of LpmP and CslZ to PG, cellulose, and chitin. CslZ or copper-loaded LpmP were incubated with PG from S. coelicolor, microcrystalline cellulose, or α-chitin for 3 h at room temperature. The supernatants, containing the unbound proteins (NB), were collected by centrifugation. The pelleted insoluble polysaccharides were washed, after which the bound (B) proteins were extracted with 4% SDS. The unbound (NB) and bound (B) proteins were analyzed using a 15% SDS-PAGE gel, revealing that LpmP binds weakly to chitin and strongly to PG. No binding was observed for CslZ. (C) LpmP facilitates hydrolysis of PG by CslZ. PG from S. coelicolor was incubated with CslZ (5 μM), CslZ^D120A^ (5 μM), LpmP (1 μM), apo-LpmP (1 μM), or combinations thereof. The difference in absorbance (Δ*A*_600_) was used as a measure for the degradation of PG. (D) Quantitative assessment of PG hydrolysis by lysozyme in the presence and absence of LpmP. PG from S. coelicolor was incubated with lysozyme (2.5 μM), LpmP (1 μM), apo-LpmP (1 μM), or combinations thereof. The difference in absorbance (Δ*A*_600_) was used as a measure for the degradation of PG. (E) Degradation of S. coelicolor PG by lysozyme (2.5 μM) with and without prior treatment with LpmP. Data points were fitted with a linear regression plot. Error bars represent the standard error of the mean of triplicate experiments.

All results indicated that CslZ and LpmP have partially overlapping roles in deposition of the cellulose-like glycan produced by CslA at the cell surface. To characterize their roles biochemically, we first produced CslZ and LpmP in Escherichia coli (Fig. 5A). The purified proteins were then tested for their ability to bind and hydrolyze a range of *β*-(1-4) glycans, including PG, cellulose, and α-chitin. CslZ did not bind to any of the substrates (Fig. 5B), in agreement with the absence of canonical carbohydrate-binding modules (see Fig. S1). However, CslZ hydrolyzed various forms of cellulose and α-chitin (Fig. S8) but also PG from S. coelicolor (Fig. 5C), showing that firm binding to these polymers is not a prerequisite for hydrolysis. To corroborate these findings, we purified a mutant form of CslZ in which the putative catalytic residue Asp120 was replaced by an alanine. Unlike the wild-type enzyme, CslZ^D120A^ was no longer able to degrade PG (Fig. 5C) or carboxymethyl cellulose (CMC) (Fig. S8B, *P = *0.010).

10.1128/mbio.00456-22.8 FIG S8 Quantitative assessment of hydrolytic activity of CslZ on cellulose and chitin substrates. (A) The amount of reducing sugar released by hydrolysis was determined using 3,5-dinitrosalicylic acid (DNSA) reagent after incubating 20 μg enzymes (CslZ, cellulase, and chitinase) with 4 mg mL^−1^ carboxymethyl cellulose (CMC), 8 mg mL^−1^ cellulose, 8 mg mL^−1^ Avicel, and 8 mg mL^−1^ α-chitin for 72 h (37°C, pH 7.5), respectively. (B) Replacement of the putative catalytic side residue D120 by an alanine residue inactivates the hydrolytic ability of CslZ (*P = *0.010), as determined by quantifying the amount of reducing sugar released following incubation of 20 μg CslZ or CslZ^D120A^ with 4 mg mL^−1^ CMC for 72 h (37°C, pH 7.5). Glucose (Sigma) was used as the standard to convert the absorbance to concentration of reducing sugars (in μM). All values were blanked against the nonenzyme control. Error bars represent the standard error mean of triplicate measurements. Download FIG S8, TIF file, 2.7 MB. Copyright © 2022 Zhong et al. 2022 Zhong et al. https://creativecommons.org/licenses/by/4.0/ This content is distributed under the terms of the Creative Commons Attribution 4.0 International license.

Interestingly, unlike CslZ, LpmP bound strongly to PG and could be detached from PG using 4% SDS (Fig. 5B). Furthermore, LpmP could also bind to α-chitin, albeit with a lower affinity than to PG (Fig. 5B). To see if the binding of LpmP to PG was functionally relevant, we also measured the ability of LpmP to facilitate PG hydrolysis (see Materials and Methods). Like CslZ, also LpmP was able to degrade PG (Fig. 5C). Furthermore, this hydrolytic activity was abolished when LpmP was used in its apo-form without the required copper cofactor (Fig. 5C). Notably, when CslZ and LpmP were both added to PG, the initial speed of hydrolysis strongly increased (Fig. 5C).

To further confirm the auxiliary role of LpmP in PG degradation *in vitro*, we tested if LpmP could also facilitate PG degradation by lysozyme. We therefore incubated PG with lysozyme in the presence or absence of LpmP (Fig. 5D). As observed with CslZ, LpmP also increased the speed of PG hydrolysis by lysozyme, which again was not observed when apo-LpmP was used (Fig. 5D). This increase in speed of hydrolysis was even more evident when LpmP and lysozyme were added sequentially: pretreatment of PG for 30 min with LpmP strongly facilitated the hydrolytic activity of lysozyme (Fig. 5E). Linear regression analysis showed a more than 2-fold increase in hydrolysis speed in the sample pretreated with LpmP, consistent with a role of LPMOs in facilitating degradation of recalcitrant polymers such as PG. Altogether, these results demonstrate that CslZ and LpmP cooperate in degradation of PG.

## DISCUSSION

Bacterial LPMOs have been implicated in a variety of functions, including virulence, nutrition, and symbiosis (35). LPMOs exert these roles by cleaving recalcitrant polysaccharides via an oxidative mechanism. In this paper, we identify for the first time an LPMO of the AA10 family that facilitates degradation of peptidoglycan. This degradation is required to expose a cellulose-like glycan on the cell surface, which plays pivotal roles in morphogenesis in *Streptomyces*. Given that LPMOs are commonly found in microbes, we anticipate that this newly identified biological role in cell wall remodeling is widespread.

**FIG 6:**
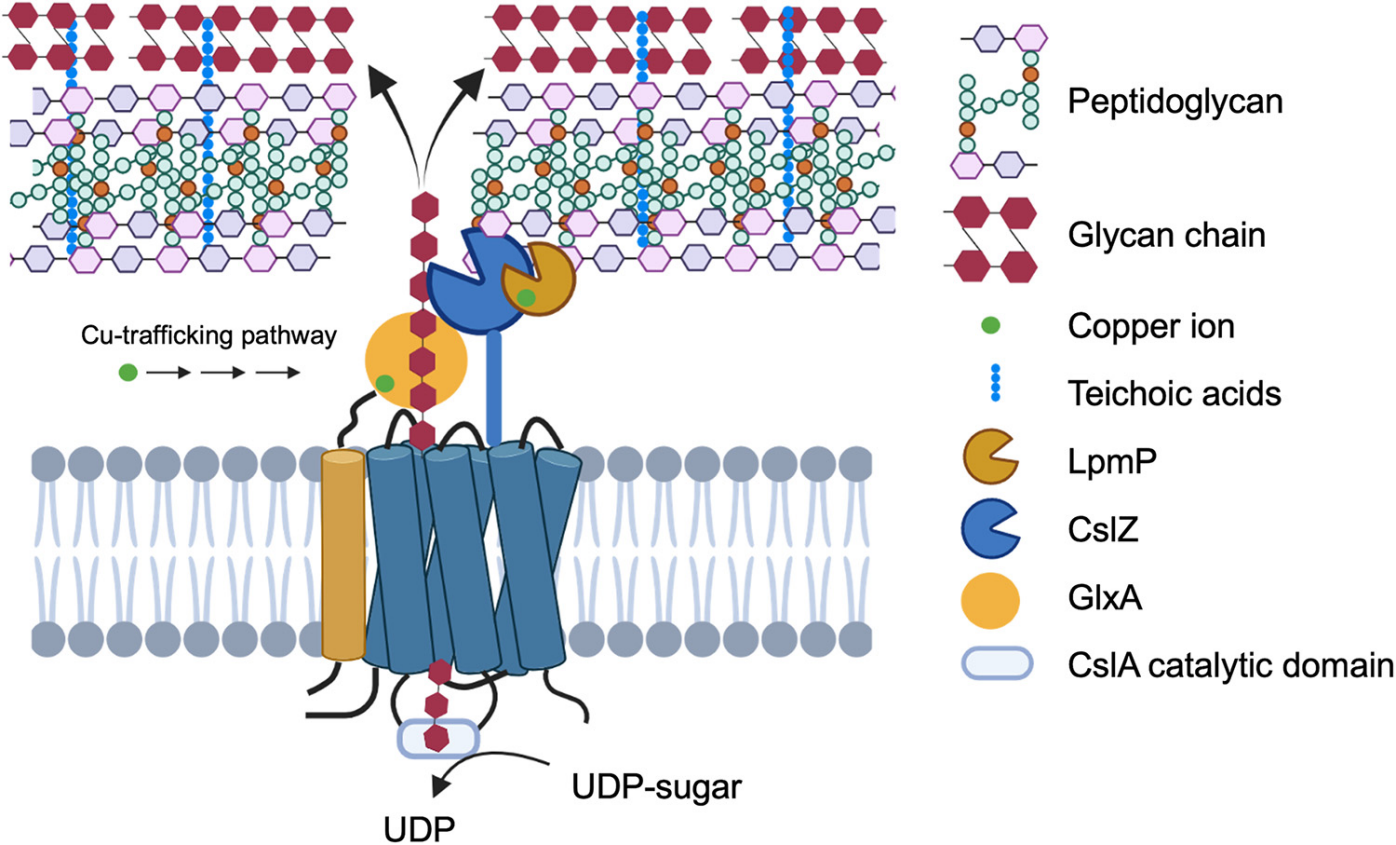
Proposed model for assembly and deposition of the apical glycan produced by CslA in *Streptomyces*. CslA utilizes UDP sugars to synthesize a glycan, which is possibly modified by the activity of the copper-containing enzyme GlxA. LpmP binds to PG and introduces random cleavages, allowing further degradation by CslZ to create a passage that allows exposure of the glycan at the cell surface. The polymer is then integrated in the cell wall, presumably via interactions involving teichoic acids (6).

Since the first report of LPMOs, these proteins have shown great potential in industrial applications with their ability to cleave polysaccharides by an oxidative mechanism (36). LPMOs perform this cleaving activity randomly in the glycan chain, thereby creating better access for more specific hydrolases to further degrade the polysaccharide. Prolific producers of LPMOs are streptomycetes, which often possess multiple LPMO-encoding genes (37–39). In fact, the best-studied representative of this group of bacteria, S. coelicolor, has 7 copies (40). It is assumed that this relatively large number is explained by the fact that these organisms thrive in environments that are rich in a variety of recalcitrant polysaccharides. Although this is certainly true, we here found that one of these LPMOs has an important role in morphological development of the producer itself. More specifically, LpmP was found to bind strongly to peptidoglycan, facilitating its degradation together with the hydrolase CslZ. Based on our results and a previous study on the S. coelicolor cell envelope architecture (6), we propose the following model. LpmP likely creates individual cuts in PG, which then becomes a substrate for further degradation by CslZ. In this manner, the combined activity of both proteins results in a localized PG degradation that is important to expose the cellulose-like glycan on the hyphal surface (Fig. 6). Characterization of the reaction products of CslZ and LpmP, by mass spectrometry or NMR, remains an important challenge for the future.

Previous work indicated that this apically localized glycan plays important roles in morphogenesis (10, 11). For instance, it is essential for the formation of reproductive aerial hyphae on solid media, indicating that without this glycan the colony is effectively sterile. Furthermore, it is also required for the formation of pellets in liquid-grown environments (12, 13). We here also find that the cellulose-like polymer provides protection against lysozyme. Notably, like the Δ*cslA* mutant, the Δ*lpmP/*Δ*cslZ* double mutant was unable to form colonies in the presence of 250 μg mL^−1^ lysozyme. This demonstrates that this polymer can serve a protective role at growing hyphal tips, as suggested earlier (11, 41). Unlike the Δ*cslA* mutant, however, the Δ*lpmP/*Δ*cslZ* double mutant could also not grow when the lysozyme concentration was reduced to 10 μg mL^−1^ lysozyme. This indicates that (i) lysozyme protection is not only conferred by the CslA-produced glycan and (ii) LpmP and/or CslZ have another function unrelated to deposition of the cellulose-like glycan. We speculate that these proteins are more broadly involved in PG remodeling and that interfering with their activities causes detrimental effects. Indeed, this could also explain why only partial complementation in lysozyme resistance was observed when both genes, expressed from a constitutive promoter, were reintroduced in the double mutant strain.

Synthesis of the cellulose-like polymer is performed by CslA in collaboration with several other proteins (12). *cslA* is part of an operon that also accommodates *glxA* and *cslZ* and which is found in almost all streptomycetes. Both CslA and GlxA are essential for formation of the functional polymer, whereby GlxA possibly modifies the nascent glycan. GlxA requires copper for its maturation, which is provided by the copper chaperone Sco (12). Indeed, the absence of this chaperone also blocks morphogenesis. Like GlxA, also LpmP is a copper-dependent enzyme. How LpmP acquires its copper is unknown, but this could also require Sco. Following synthesis of the glycan by CslA/GlxA, the polymer needs to traverse the thick PG layer. Based on our data, we propose that localized PG hydrolysis by LpmP and the promiscuous hydrolase CslZ is necessary and sufficient to create a channel through the PG layer to ensure that the glycan produced by CslA becomes localized exterior of the PG (Fig. 6). This is consistent with the observation that the polymer produced by CslA was absent from hyphal tips in strains lacking both *lpmP* and *cslZ*. We expect that PG hydrolysis is confined to regions in proximity of the sites where CslZ and LpmP are secreted. As a lipoprotein, CslZ is tightly associated with the membrane limiting its ability to diffuse. In contrast, LpmP can theoretically freely diffuse in the cell wall matrix. However, movement is likely limited due to the strong binding ability of LpmP to PG. We therefore expect that LpmP and CslZ will mainly act close to their secretion sites. In this manner, the cell can retain its integrity, even in strains producing large quantities of these proteins.

Biosynthesis of cellulose has been best studied in the Gram-negative bacterium E. coli, where cellulose is produced by the BcsA/BcsB complex. Extrusion of the cellulose microfibrils in the environment is mediated by the conserved BcsC protein, which binds to peptidoglycan while also forming an exit pore through the outer membrane (42). However, how cellulose is crossing the peptidoglycan layer is not described for any of the well-studied cellulose systems. Perhaps crossing of the PG layer in Gram-negative bacteria is possible without specific hydrolases given that the PG layer is relatively thin in these organisms.

In conclusion, our work identifies a set of proteins that are the likely candidates to facilitate traversing of the cellulose-like glycan through the thick PG layer. The involvement of an LPMO associates this class of proteins with PG remodeling, which is an important step in any growing bacterial cell. We therefore believe that this work will open important new avenues to further understand PG remodeling, while also providing new opportunities for drug discovery aimed at identifying molecules that interfere with this process.

## MATERIALS AND METHODS

### Bacterial strains and culture conditions.

All strains used in this study are listed in Table 2. Mannitol soy flour (MS) agar plates were used for collection of spores and for conjugation experiments, while phenotypic analyses were performed on solid R5 medium (43). To study the morphology in liquid environments, freshly prepared *Streptomyces* spores were inoculated in 100 mL tryptic soy broth sucrose (TSBS) medium in 250 mL unbaffled Erlenmeyer flasks equipped with metal coils at a final concentration of 10^6^ CFU mL^−1^. Cultures were grown at 30°C while shaking at 200 rpm min^−1^.

**TABLE 2:** Strains used in this study

Strain	Description	Reference
*Streptomyces*		
S. coelicolor M145	Wild-type Streptomyces coelicolor A3(2) strain	Laboratory collection
pM145	M145 containing pSET152	This work
Δ*cslA*	M145 lacking the *cslA* gene	9
Δ*cslZ*	M145 lacking the *cslZ* gene	This work
Δ*lpmP*	M145 lacking the *lpmP* gene	This work
Δ*cslZ/*Δ*lpmP*	Double mutant lacking *cslZ* and *lpmP*	This work
M145 + pXZ2	S. coelicolor M145 containing pXZ2 that constitutively expresses *cslZ* from the *gapAp* promoter	This work
M145 + pXZ3	S. coelicolor M145 containing pXZ3 that constitutively expresses *lpmP* from the *gapAp* promoter	This work
M145 + pXZ4	S. coelicolor M145 containing pXZ4 that constitutively expresses *cslZ* and *lpmP* from the *gapAp* promoter	This work
Δ*cslZ* + hpXZ2	S. coelicolor Δ*cslZ* containing hpXZ2 that constitutively expresses *cslZ* from the *gapAp* promoter	This work
Δ*lpmP* + pXZ3	S. coelicolor Δ*lpmP* containing pXZ3 that constitutively expresses *lpmP* from the *gapAp* promoter	This work
Δ*cslZ/*Δ*lpmP* + hpXZ4	S. coelicolor Δ*cslZ/*Δ*lpmP* containing hpXZ4 that constitutively expresses *cslZ* and *lpmP* from the *gapAp* promoter	This work
E. coli		
DH5α	F- Φ80lacZDM15, Δ(lacZYA-argF), for cloning	Laboratory collection
BL21(DE3)	*Lon*, *ompT*, *gal*, λDE3, for protein expression	Laboratory collection
ET12567(pUZ8002)	*dam- dcm- hsdS*, RP4 transfer gene	44
BTH101	F-, *cya-99*, *araD139*, *galE15*, *galK16*, *rpsL1* (*Str^r^*), *hsdR2*, *mcrA1*, *mcrB1*	Euromedex

E. coli strains DH5α and BL21(DE3) were used for routine cloning purposes and for expression of proteins, respectively. E. coli ET12567 harboring pUZ8002 was used to obtain unmethylated plasmid DNA and for conjugation of plasmids to *Streptomyces* (44). For the bacterial two-hybrid analyses, BTH101 was used (Euromedex). All E. coli strains were grown at 37°C in LB medium supplemented with the appropriate antibiotics, if necessary.

### Construction of plasmids and strains.

For expression of CslZ in E. coli, genomic DNA of S. coelicolor was used as the template to amplify nucleotides 97 to 999 of the coding region of *cslZ* (also called SCO2838) using primers cslZ-F and cslZ-R (see Table 3 for all primers used in this study), in which the original signal peptide (1 to 96 nucleotides) was removed. The amplified sequence was cloned as an NcoI-HindIII fragment into pET28a (Novagen), yielding pXZ1. To create pD120A (for expressing CslZ^D120A^), site-directed mutagenesis was used with pXZ1 and primers mCslZ-F1/mCslZ-R1. Plasmids were subsequently introduced into E. coli BL21(DE3) by transformation (45). The plasmid, pET26b-LPMO, used to express LpmP in E. coli BL21(DE3) was a gift from Jonathan A. R. Worrall (University of Essex).

**TABLE 3:** List of primers used in this study

Primer name	Primer sequence (5′ to 3′)
CslZ-F	CATGCCATGGCGGGCGCCGGGATCACCCAG
CslZ-R	CCCAAGCTTGCCCCTGCGCCAGTTTCAAGGCGTACTCG
cslZ-flag-F	GGGAATTCCATATGTCCAGGAGGCGGGCTGC
cslZ-flag-R	CCGGAATTCTTACTTGTCGTCATCGTCTTTGTAGTCACCAGAACCACCAGAACCCCCCTGCGCCAGTTTCAAGGC
mCslZ-F1	ATCCCGCACCGCGCCTGCGGCCAGTACTCC
mCslZ-R1	GTTGTAGAGGACGAGCAGCGCGGTGCGGCCG
gapA-F(BamHI)	CGCGGATCCGTCCTCGCCGACGAGGCCTC
gapA-R	GGGAATTCCATATGGAACCGATCTCCTCGTTGGTAC
2838-F	GGGAATTCCATATGTCCAGGAGGCGGGCTGCGTC
2838-R	CCGGAATTCTTACCCCTGCGCCAGTTTCAAG
gapA-F(XbaI)	TGCTCTAGAGTCCTCGCCGACGAGGCCTC
2833-F	GGAATTCCATATG ATGCGCACAAGGACCAAGTTG
2833-R	CGCGGATCCTCAGAAGGTGACGTCCGAGC
CBest-spacer-F	CATGCCATGGGCAGAGCGTGGAGGGGCCCAGTTTTAGAGCTAGAAATAGC
CBest-R	CAGTGGTTATGCTAGTTACGCCTACGTA
hyg-F	GATATCGATCGGCGGGGCCTGGCGGC
hyg-R	GATATCGATCGGCGGGGCCTGGGACG
Th2833-F	GCCTCTAGAACACGGCTACACCGACCTGCC
Th2833-R	CGGGAATTCTCAGAAGGTGACGTCCGAGCAGGC
Th2838-F	GCCTCTAGAAGCGGGCGCCGGGATCACCC
Th2838-R	CGGGAATTCTCACCCCTGCGCCAGTTTCAAGGCGTACTCG
Th2833_FL_-F	GCCTCTAGAAATGCGCACAAGGACCAAGTTGTAC
Th2833_FL_-R	CGGGAATTCTCAGAAGGTGACGTCCGAGC
Th2838_FL_-F	GCCTCTAGAAATGTCCAGGAGGCGGGCTGC
Th2838_FL_-R	CGGGAATTCTTACCCCTGCGCCAGTTTCAAGGC

To constitutively express CslZ in S. coelicolor, the *gapAp* promoter of SCO1947 and coding sequence of *cslZ* were amplified from genomic DNA of S. coelicolor using primers gapA-F(BamHI)/gapA-R and 2838-F/2838-R, respectively. The amplified products were then cut with the restriction enzymes BamHI-NdeI (*gapAp*) and NdeI-EcoRI (*cslZ*), after which the digested fragments were ligated together in pSET152 (46) that had been cut with BamHI and EcoRI, yielding pXZ2. To create a FLAG-tagged version of CslZ, a similar procedure was used but now using primers cslZ-flag-F/cslZ-flag-R to amplify *cslZ*. The amplified fragment was then cloned together with the *gapAp* promoter into pSET152, yielding pXZ2f.

For constitutive expression of LpmP in *Streptomyces*, the *gapAp* promoter and coding sequence of SCO2833 were amplified from genomic DNA of S. coelicolor using primers gapA-F(XbaI)/gapA-R and 2833-F/2833-R, respectively. The amplified products were then cut using the restriction enzymes XbaI-NdeI (*gapAp*) and NdeI-BamHI (SCO2833) and ligated into pSET152 that had been digested with XbaI and BamHI, yielding pXZ3.

The construct used to overexpress both CslZ and LpmP, termed pXZ4, was generated by isolating the *gapAp-cslZ* fragment from pXZ2 using BamHI and EcoRI and inserting this fragment into pXZ3 plasmid digested with the same enzymes. For complementation studies, we also created variants of pXZ2 and pXZ4 carrying a hygromycin resistance cassette, termed hpXZ2 and hpXZ4, respectively. To this end, the hygromycin resistance gene was amplified from pIJ82 (kindly provided by B. Gust) using primers hyg-F/hyg-R. The PCR product was digested with EcoRV and inserted into pXZ2 and pXZ4 which had been digested with SacI. All constructs were subsequently introduced in S. coelicolor via conjugation (44).

The *cslZ* null mutant in S. coelicolor was constructed using plasmid pΔcslZ as described previously (12). Inactivation of the *lpmP* gene was achieved by creating a stop codon at nucleotide position 406 through the single-nucleotide–resolution genome editing system pCRISPR-cBEST (47). Briefly, a fragment was amplified from the pCRISPR-cBEST plasmid with primers CBest-spacer-F and CBest-R, thereby introducing the *lpmP*-targeting spacer. This PCR product was then cloned into pCRISPR-cBEST via NcoI and SnaBI to generate plasmid pXZ5. After conjugation, individual exconjugants were randomly picked and streaked on MS agar plates supplemented with 20 μg mL^−1^ thiostrepton. Colonies were then streaked again on MS plates without any antibiotics, after which single colonies were picked and inoculated in 2 mL TSBS medium. After 3 days, genomic DNA was isolated and the coding sequence of SCO2833 was PCR-amplified using primers 2833-F/2833-R, followed by sequencing of the PCR product. The spacer used to create the mutation was generated using CRISPY-web (48) and is listed in Table 3. All mutants were verified by sequencing.

For the bacterial two-hybrid analyses, *lpmP* and *cslZ* were amplified from genomic DNA of S. coelicolor using primers Th2833_FL_-F/Th2833_FL_-R and Th2838_FL_-F/Th2838_FL_-R, respectively. Likewise, fragments of these genes were amplified encoding LpmP and CslZ without their signal sequences, using primers Th2833-F/Th2833-R and Th2838-F/Th2838-R, respectively. The amplified *lpmP* and *cslZ* fragments were subsequently cloned into pKT25 or pUT18C plasmids (49) as XbaI/EcoRI fragments, yielding pXZ6, pXZ7, pXZ8, and pXZ9 (see Table 4). All constructs were verified by DNA sequencing and used for cotransformations to E. coli *cya* BTH101 competent cells.

**TABLE 4:** Plasmids used in this work

Plasmid	Description	Reference
pET26b-LPMO	pET26b containing nucleotides 88–606 of the S. lividans *lpmP* gene	15
*pΔcslZ*	pWHM3 derivative containing the flanking regions of the S. lividans *cslZ* gene (SLI3189) interspersed by the apramycin*-loxP* cassette	12
pXZ1	pET28a plasmid containing nucleotides 97–999 of *cslZ*	This work
pXZ2	pSET152 plasmid containing *cslZ* expressed from the constitutive *gapAp* promoter	This work
pXZ3	pSET152 plasmid containing *lpmP* expressed from the constitutive *gapAp* promoter	This work
pXZ4	pSET152 plasmid containing *cslZ* and *lpmP* expressed from the constitutive *gapAp* promoter	This work
pXZ5	pCRISPR-cBEST plasmid containing the spacer targeting *lpmP*	This work
pD120A	pXZ1 derivative in which nucleotide 359 of *cslZ* was substituted (from A to C)	This work
pXZ2f	pSET152 derivative containing *cslZ-flag* expressed from the constitutive *gapAp* promoter	This work
hpXZ2	pXZ2 derivative carrying a hygromycin resistance cassette	This work
hpXZ4	pXZ4 derivative carrying a hygromycin resistance cassette	This work
pKT25	pSU40 derivative expressing the T25 fragment of CyaA (amino acids 1–224)	49
pUT18C	pUC19 derivative expressing the T18 fragment of CyaA (amino acids 225–339)	49
pKT25-zip	pKT25 plasmid containing a 35-amino-acids-long leucine zipper region of yeast protein GCN4	49
pUT18C-zip	pUT18C plasmid containing a 35-amino-acids-long leucine zipper region of yeast protein GCN4	49
pXZ6	pKT25 containing nucleotides 88 to 606 of *lpmP*	This work
pXZ7	pKT25 containing nucleotides 1–606 of *lpmP*	This work
pXZ8	pUT18C containing nucleotides 97–999 of *cslZ*	This work
pXZ9	pUT18C containing nucleotides 1–999 of *cslZ*	This work

### Bioinformatic analysis.

To investigate the glycoside hydrolase (GH) family that CslZ belongs to, BLASTP (http://blast.ncbi.nlm.nih.gov) was used (50). The Carbohydrate-Active Enzymes database (CAZy) was used to investigate similarities of CslZ to known members of the GH6 family (28). Representative GH6 proteins were selected and included Thermobifida fusca Cel6A (*Tf*Cel6A), Thermobifida fusca Cel6B (*Tf*Cel6B), Teredinibacter turnerae CelAB, and Cel6H from an uncultured bacterium. GH6 domains contained in these proteins were predicted by InterPro (https://www.ebi.ac.uk/interpro/), and alignments of these domains was performed using Cluster Omega (https://www.ebi.ac.uk/Tools/msa/clustalo/). The phylogenetic analysis of CslZ was done with Phylogeny.fr (51) using a collection of 11 hydrolases belonging to the GH6 family, including XpCel6A (52), CelAB (53), CbhA (54), XylK2 (55), CbhII (56), TfCel6B (57), CenA (58), EGI (59), TfCel6A (60), McenA (61), and TbCel6A (62). This selection of GH6 hydrolases was made on the availability of experimental data on their substrates.

### Microscopy.

Pellets were imaged using a Zeiss Axiomicroscope equipped with an Axiocam 105 camera as described previously (33). β-(1-4) Glycans were stained with calcofluor white (Sigma) as described previously (9, 11). Stack acquisition was done on a Zeiss LSM900 Airyscan 2 microscope. All fluorescent images were imaged with the same setting (laser intensity: 3.5%, pinhole: 47 μm, master gain: 750V, digital offset: −15, and digital gain: 1.0). For quantitatively comparing fluorescence, the measure region with the size of 15 μm by 15 μm squares at hyphal tips was used. Fluorescence was measured using ImageJ software (version 2.0.0/1.53c/Java 1.8.0_172/64-bit) (63).

### Lysozyme and antibiotic sensitivity assays.

Lysozyme sensitivity assays were performed by plating approximately 1,000 spores of each strain on Difco nutrient agar plates either not supplemented or supplemented with 0.25 mg mL^−1^ lysozyme (from chicken egg white, ≥40,000 units mg^−1^, Sigma). After 48 h of growth, the total number of colonies was counted. For every strain, the number of colonies on the plate with lysozyme was divided by the number of colonies on the plate without lysozyme as an estimate for lysozyme sensitivity. Antibiotic sensitivity assays were performed with discs diffusion assays using 50 μg mL^−1^ ampicillin or penicillin G.

### Expression and purification of CslZ and LpmP.

The LpmP protein was produced in BL21(DE3) and purified as described previously (15), except that the purified protein was stored in buffer C containing 25 mM Tris–HCl and 200 mM NaCl (pH 7.5).

To purify CslZ and CslZ^D120A^, E. coli cells harboring plasmid pXZ1 or pD120A (Table 4) were cultured at 37°C to an optical density at 600 nm (OD_600_) of 0.6 in LB medium containing 50 μg mL^−1^ kanamycin. Then, expression was induced by adding 1 mM isopropyl β-d-1-thiogalactopyranoside and cells were grown at 20°C for 18 h. The induced cells were lysed by sonication in binding buffer (25 mM Tris–HCl, 200 mM NaCl [pH 7.5]), and after centrifuging the lysate was loaded on a Co^2+^-chelating column equilibrated with binding buffer. Ten column volumes of binding buffer and 10 mL of elution buffer (25 mM Tris–HCl, 200 mM NaCl, 10 mM imidazole [pH 7.5]) were used to wash and elute CslZ, respectively. The protein was finally purified by gel filtration using a Superdex 200 Increase 10/300 GL column (GE Healthcare) equilibrated with binding buffer. Sample fractions were analyzed by SDS-PAGE. If necessary, fractions were concentrated to 5 mg mL^−1^ with the 10 kDa molecular weight cutoff concentrator (Millipore).

### Preparation of Cu-loaded LpmP.

To load copper on LpmP, copper (II) sulfate (Sigma) was added to reach a 2× mole equivalent of purified LpmP. After incubation for 15 min at room temperature, the excess copper was removed by applying the protein samples to a Superdex 200 Increase 10/300 GL column equilibrated with buffer 25 mM Tri-HCl (pH 7.5). After collection, fractions were concentrated as described above.

### Isolation of peptidoglycan from S. coelicolor.

PG was isolated from the wild-type strain as described previously (6) with the exception that the HF treatment was omitted.

### Substrate binding assay.

Binding of LpmP and CslZ to different polymers was essentially performed as described previously (15, 64), with the following modifications. Briefly, 50 μg of purified Cu-LpmP or CslZ protein was incubated for 3 h at room temperature with 10 μL PG from Streptomyces coelicolor, 5 mg α-chitin from shrimp shells (Sigma), or 5 mg microcrystalline cellulose (Sigma) in 100 μL 25 mM Bis-Tris HCl buffer (pH 6.2). The supernatant was then separated from the polymers by centrifugation for 20 min at 14,000 × *g* and kept as the fraction containing unbound protein. The polymers were then washed two times with wash buffer (25 mM Bis-Tris HCl [pH 6.2]) to remove weakly bound proteins. Strongly bound proteins were extracted from the polymers by adding 4% SDS solution and incubating the samples for 1 h at room temperature. Samples were then analyzed with SDS-PAGE using a 15% gel.

### Quantitative assessment of hydrolytic activity.

The quantitative analysis of the hydrolytic activity of CslZ was essentially performed as described previously (65) with the following modification. Reactions were carried out in 20 mM Tris buffer (pH 7.5) supplemented with 4 mg mL^−1^ carboxymethyl cellulose (CMC) sodium salt (Sigma), 8 mg mL^−1^ microcrystalline cellulose (Sigma), 8 mg mL^−1^ Avicel PH-101 (Sigma), or 8 mg mL^−1^ α-chitin (from shrimp shells, Sigma). For each reaction, 20 μg CslZ or CslZ^D120A^ was used and the mixtures were incubated at 37°C while shaking at 250 rpm min^−1^. As a control, a commercial cellulase (from Aspergillus niger, Megazyme) and chitinase (from Streptomyces griseus, ≥200 units/mg, Sigma) were used. After incubation for 72 h, the reaction mixture was centrifuged, and the reducing sugars in the supernatant were detected using the 3,5-dinitrosalicylic acid (DNSA) reagent in a microtiter plate reader (66). All measurements are the average of three replicates.

Quantitative assessment of LpmP and CslZ activity on PG was performed using a turbidimetric analysis (67). Briefly, 2.5 μM lysozyme (Sigma), 5 μM purified CslZ or CslZ^D120A^, 1 μM Cu-LpmP, or mixtures thereof were incubated with 60 μL PG from Streptomyces coelicolor in 600 μL reaction buffer (25 mM Bis-Tris HCl, 100 mM NaCl, 1 mM ascorbic acid [pH 6.2]) in a Sarstedt polystyrene cuvette with 45 mm path length and sealed with Parafilm. All samples were prepared on ice. The initial absorbance at 600 nm (*A*_600_) was measured using a BIO-RAD SmartSpec 3000 spectrophotometer, after which the cuvettes were incubated at 37°C while shaking. Subsequently, the *A*_600_ values for each reaction were measured at different time points (up until 180 min). The difference in absorbance (Δ*A*_600_), calculated by subtracting the initial *A*_600_ from the *A*_600_ at each time point, was used to quantify PG degradation. All reactions were performed in triplicate.

### Preparation of mycelium fractions and immunoblotting.

Preparation of mycelial membrane/cytoplasmic fractions and immunoblotting were essentially performed as described previously (13). For the detection of FLAG-tagged CslZ, the monoclonal ANTI-FLAG M2 antibody (Sigma) and an anti-mouse lgG-alkaline phosphatase (Sigma) were used as the primary and secondary antibody, respectively. Images were collected using an Epson Perfection V37 scanner.

### Bacterial two-hybrid assay.

The bacterial two-hybrid analyses were essentially performed as described. Following cotransformation, ampicillin- and kanamycin-resistant transformants were selected and grown overnight. Then, 5 μL of the overnight cultures were spotted on M63/maltose minimal medium-agar plate supplemented with 40 μg mL^−1^ X-gal, 0.5 mM IPTG (isopropyl-β-d-thiogalactopyranoside), 50 μg mL^−1^ ampicillin, and 25 μg mL^−1^ kanamycin. After growth for 4 days at 37°C, protein interactions were evaluated by assessing the color of the colonies. Plates were scanned using an Epson Perfection V37 scanner.

### Statistical analysis.

GraphPad Prism software (version 8.0.2) was used for statistical analyses. For pairwise comparisons, paired *t* tests were done.
