# Supplementary figures and images for: Role for a Lytic Polysaccharide Monooxygenase in Cell Wall Remodeling in Streptomyces coelicolor
Source: mBio. 2022 Mar 31;13(2):e00456-22. doi: 10.1128/mbio.00456-22 (PMC9040799; doi:10.1128/mbio.00456-22)

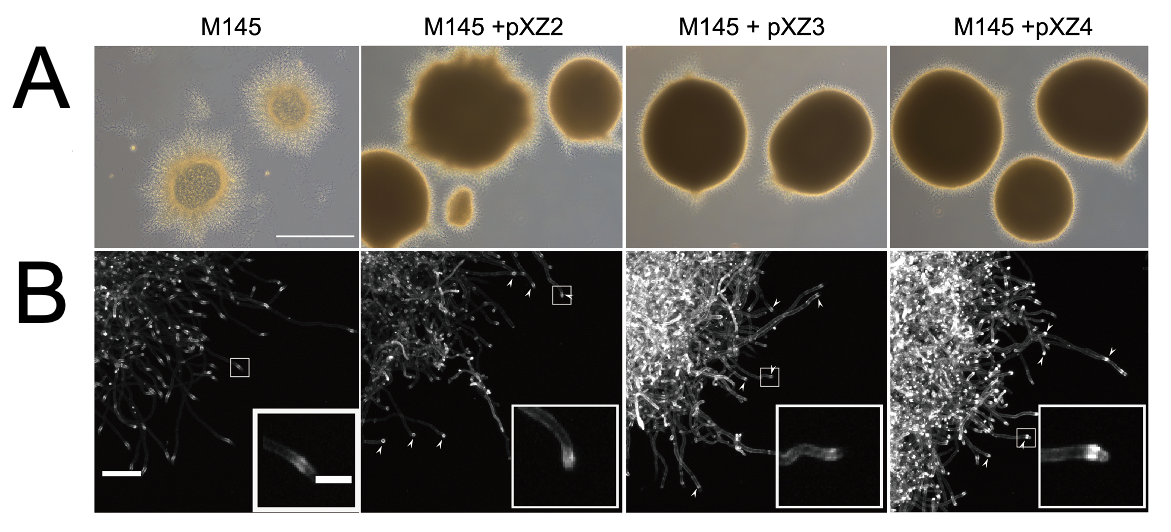

Supplement: FIG S3 [file mbio.00456-22-sf003.tif]

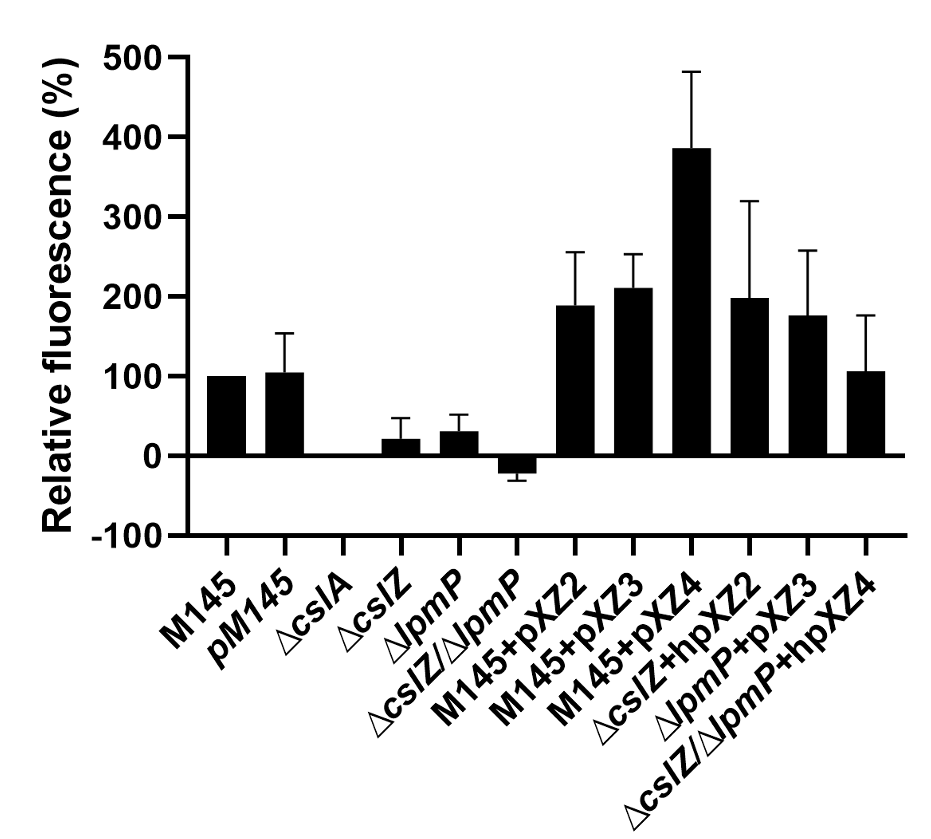

Supplement: FIG S4 [file mbio.00456-22-sf004.tif]

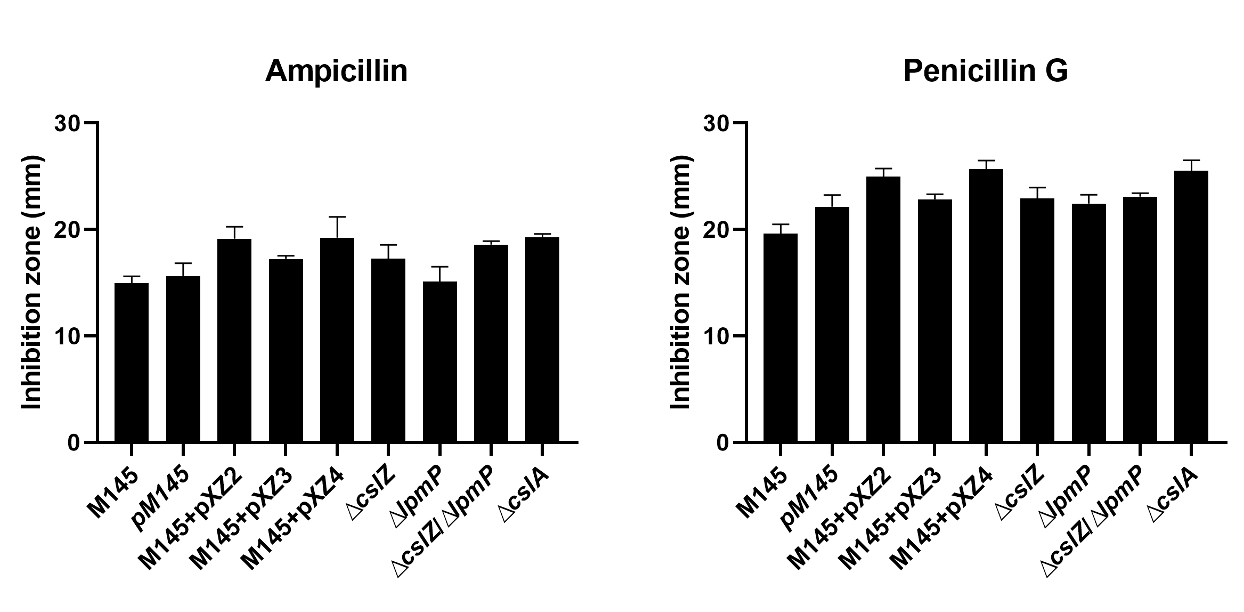

Supplement: FIG S5 [file mbio.00456-22-sf005.tif]

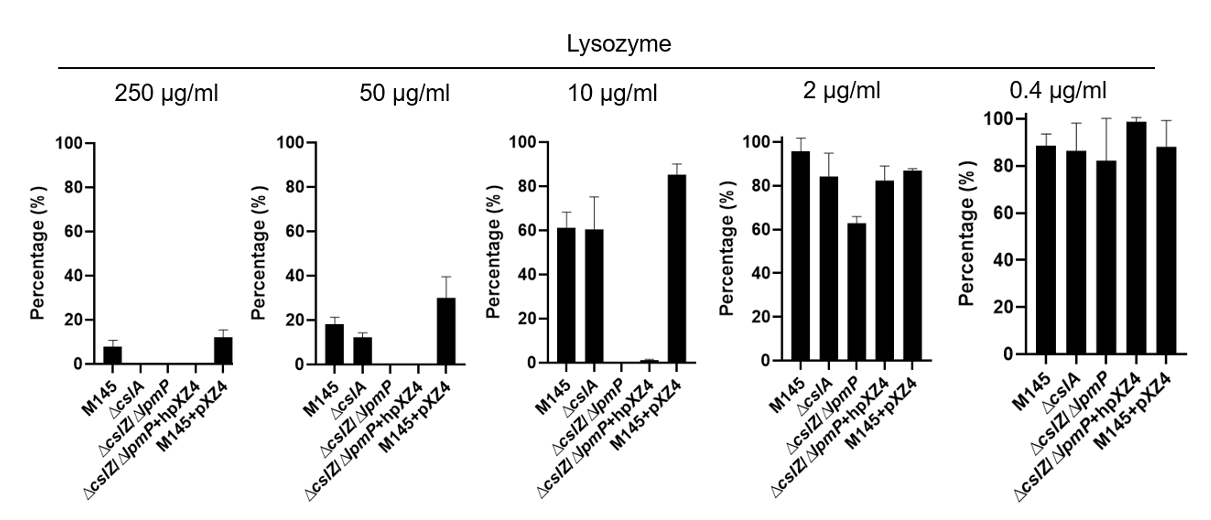

Supplement: FIG S6 [file mbio.00456-22-sf006.tif]

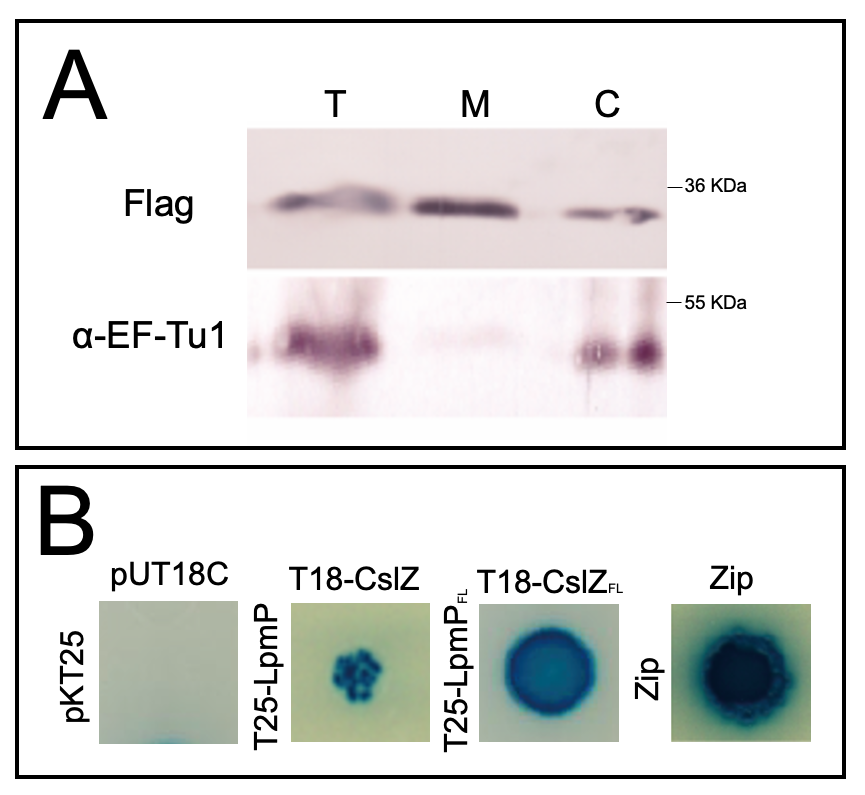

Supplement: FIG S7 [file mbio.00456-22-sf007.tif]

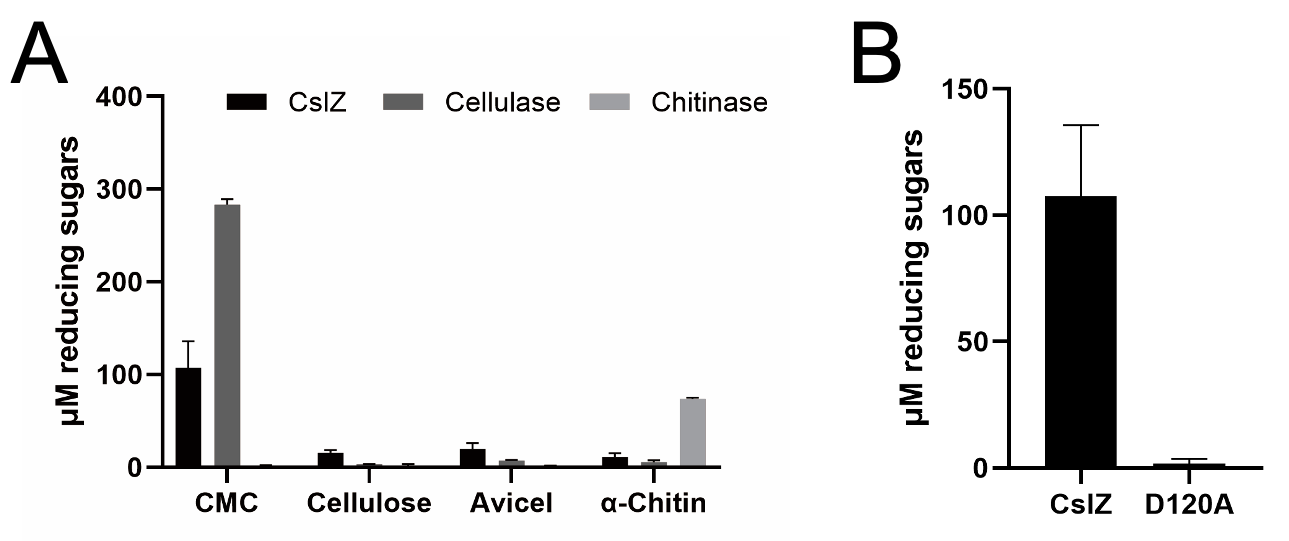

Supplement: FIG S8 [file mbio.00456-22-sf008.tif]
